# Antioxidant Phytochemicals for the Prevention and Treatment of Chronic Diseases

**DOI:** 10.3390/molecules201219753

**Published:** 2015-11-27

**Authors:** Yu-Jie Zhang, Ren-You Gan, Sha Li, Yue Zhou, An-Na Li, Dong-Ping Xu, Hua-Bin Li

**Affiliations:** 1 Guangdong Provincial Key Laboratory of Food, Nutrition and Health, Department of Nutrition, School of Public Health, Sun Yat-Sen University, Guangzhou 510080, China; zhyujie3@mail2.sysu.edu.cn (Y.-J.Z.); zhouyue3@mail2.sysu.edu.cn (Y.Z.); lianna28@163.com (A.-N.L.); dongpxu@163.com (D.-P.X.); 2 School of Biological Sciences, The University of Hong Kong, Hong Kong, China; ganry@connect.hku.hk; 3 School of Chinese Medicine, The University of Hong Kong, Hong Kong, China; lishasl0308@163.com

**Keywords:** antioxidant phytochemicals, health benefits, mechanism, cardiovascular disease, cancer

## Abstract

Overproduction of oxidants (reactive oxygen species and reactive nitrogen species) in the human body is responsible for the pathogenesis of some diseases. The scavenging of these oxidants is thought to be an effective measure to depress the level of oxidative stress of organisms. It has been reported that intake of vegetables and fruits is inversely associated with the risk of many chronic diseases, and antioxidant phytochemicals in vegetables and fruits are considered to be responsible for these health benefits. Antioxidant phytochemicals can be found in many foods and medicinal plants, and play an important role in the prevention and treatment of chronic diseases caused by oxidative stress. They often possess strong antioxidant and free radical scavenging abilities, as well as anti-inflammatory action, which are also the basis of other bioactivities and health benefits, such as anticancer, anti-aging, and protective action for cardiovascular diseases, diabetes mellitus, obesity and neurodegenerative diseases. This review summarizes recent progress on the health benefits of antioxidant phytochemicals, and discusses their potential mechanisms in the prevention and treatment of chronic diseases.

## 1. Introduction

Chronic diseases such as cardiovascular diseases (CVD), diabetes and cancers are global health problems, and cause death and disability to millions of people. It has been demonstrated that fruits, vegetables and grains exert a protective effect against the development of these chronic diseases [1,2,3,4]. This protective role can be mainly attributed to the phytochemicals in them, which are defined as bioactive non-nutrient compounds in fruits, vegetables, grains, and other plants [5]. So far, about 10,000 phytochemicals have been identified, and still a large percentage remains unknown. These identified phytochemicals include tannins, flavones, triterpenoids, steroids, saponins, and alkaloids [6]. The protective role of phytochemicals may be associated with their antioxidant activity, since overproduction of oxidants (reactive oxygen species and reactive nitrogen species) in the human body is involved in the pathogenesis of many chronic diseases. In order to provide an extensive and deep understanding of antioxidant phytochemicals in human health and diseases, this review, summarizes and discusses their sources and health benefits, with special attention paid to the potential mechanisms of action in the prevention and treatment of chronic diseases.

## 2. Sources of Antioxidant Phytochemicals

Antioxidant phytochemicals exist widely in fruits, vegetables, cereal grains, edible macrofungi, microalgae, and medicinal plants [7,8,9]. Common fruits, such as berries, grape, Chinese date, pomegranate, guava, sweetsop, persimmon, Chinese wampee and plum are rich in antioxidant phytochemicals [10,11,12]. In addition, wild fruits, such as the fruits of *Eucalyptus robusta*, *Eurya nitida*, *Melastoma sanguineum*, *Melaleuca leucadendron*, *Lagerstroemia indica*, *Caryota mitis*, *Lagerstroemia speciosa* and *Gordonia axillar* also have high antioxidant capacities and total phenolic contents [13]. Besides, fruits wastes (peel and seed) also contain high contents of antioxidant phytochemicals, including catechin, cyanidin 3-glucoside, epicatechin, gallic acid, kaempferol, and chlorogenic acid [14]. Some vegetables, such as Chinese toon bud, loosestrife, penile leaf, cowpea, caraway, lotus root, sweet potato leaf, soy bean (green), pepper leaf, ginseng leaf, chives, and broccoli are found to have high antioxidant capacities and total phenolic contents [15]. Among cereal grains pigmented rice, such as black rice, red rice and purple rice, possess high contents of antioxidant phytochemicals (flavones and tannins) [16]. Among selected Chinese medicinal plants the highest antioxidant capacities and phenolic contents are found in *Dioscorea bulbifera*, *Eriobotrya japonica*, *Tussilago farfara* and *Ephedra sinica* [17], and several flowers including edible and wild ones also have high contents of antioxidant phytochemicals [18].

Polyphenols and carotenoids are the two main kinds of antioxidant phytochemicals, and they contribute the most to the antioxidant properties of foods/plants. For example, β-carotene, quercetin, myricetin and kaempferol are the main antioxidant phytochemicals found in Cape gooseberry [19], and anthocyanins and ellagitannins are the major antioxidant compounds among the phytochemicals of strawberry [20]. In addition, flavonoids isolated from *Euterpe oleracea* pulp present an important antioxidant activity measured by oxygen radical absorbance capacity [21]. Natural polyphenols are the most abundant antioxidants in human diets, and their radical scavenging activities are related to substitution of hydroxyl groups in the aromatic rings of phenolics [22]. The plant variety, geographic region, growing season, and storage can all influence the concentrations of polyphenols in food [23]. Dietary polyphenols could be classified into five classes: flavonoids, phenolic acids, stilbenes, tannins and coumarins. Flavonoids can be further categorized as flavonols, flavones, flavanols, flavanones, anthocyanidins, and isoflavonoids [24]. Total phenolic content and total antioxidant activity in phytochemical extracts of different fruits may have a direct relationship. When the fruits contain higher total phenolic contents, they possess stronger antioxidant activity [25]. For example, the scavenging activity of grape seed extract against ABTS radical was strongly linked with the level of phenolic compounds [26]. Carotenoids are a group of phytochemicals that are responsible for the yellow, orange and red colors of the foods. α-Carotene, β-carotene, lycopene, lutein and cryptoxanthin are the main carotenoids in the diet and human body, and fruits and vegetables are the major sources of carotenoids in human diet. For example, tomato is rich in lycopene, which is also responsible for its characteristic red color.

## 3. Prevention and Treatment of Antioxidant Phytochemicals for Several Chronic Diseases

Overproduction of oxidants in human body can cause an imbalance and lead to oxidative damage to large biomolecules such as lipids, DNA, and proteins. This damage is responsible for the pathogenesis of several human diseases, including CVD, certain types of cancers, and aging [27,28]. Thus, antioxidant phytochemicals could play an important role in the prevention and treatment of chronic diseases [26,29]. Phytochemicals are demonstrated to have antioxidant abilities not only *in vitro* but in human studies*.* Consumption of fruits and vegetables with high contents of antioxidant phytochemicals is proven to increase the antioxidant capacity of serum/plasma. For example, the total antioxidant capacity of serum was increased significantly following consumption of red wine, strawberries, vitamin C or spinach in elderly women, and the plasma vitamin C levels and serum urate levels also increased significantly. However, the increased vitamin C and urate levels could not fully account for the increased total antioxidant capacity in serum [30]. The results were in agreement with results from another study, which found that plasma antioxidant capacity was significantly increased by consuming 10 servings of fruit and vegetables each day for 15 days [31]. This increase could not be explained by the increase of α-tocopherol concentration in the plasma. Perhaps, the increased total antioxidant capacity could be explained by increased polyphenols because a study found that 19 of the 25 anthocyanins present in the blueberries could be detected in human serum. The appearance of total anthocyanins in the serum contributed to an increase in serum antioxidant capacity [32]. In addition, polyphenols may enhance the total oxidant-scavenging capacities of human blood by binding to red blood cells [33]. Furthermore, the antioxidant activity of apples may mainly come from phenolics and flavonoids because the vitamin C in apples with skin accounts for only 0.4% of the total antioxidant activity. The additive and synergistic effects of phytochemicals in fruit and vegetables could be responsible for their potent antioxidant activities [34,35].

Chronic inflammation is another important factor that may cause or assist in the pathogenesis of many chronic diseases including CVD, cancers, and type 2 diabetes (T2D) [36,37,38]. Most antioxidant phytochemicals have been found to have anti-inflammatory action. Phytochemicals including resveratrol, anthocyanins, and curcumin, can reduce inflammation via inhibition of prostaglandin production and nuclear factor-κB activity, enzyme inhibition, as well as increase of cytokine production [21,39]. Usually, antioxidant phytochemicals possess strong antioxidant and free radical scavenging abilities as well as anti-inflammatory action, which are also the basis of other bioactivities and health benefits [9,40].

### 3.1. Protective Action on Cardiovascular Diseases

Cardiovascular disease is the leading cause of death and disability in developed countries [41]. Epidemiological studies have shown that flavonoids are linked to reduced incidence or mortality from CVD among adults in Europe and the United States [42,43]. The etiology of CVD is very complex, and overproduction of oxidants is one of the main pathogenic factors. Oxidative damage can cause endothelial cell injuries and deleterious vasodilator effects. It has been shown that antioxidant polyphenols could modify molecular events towards an improvement in endothelial function, and therefore play an important role in the prevention of CVD. Flavonoids from *Euterpe oleracea* pulp showed atheroprotective effects *in vitro* [21]. Another study indicated that total flavonoids of *Flos chrysanthemi* showed vasodilating effects and protected vasodilator reactivity from oxidative stress, which was mediated by endothelium-derived hyperpolarizing factor [44]. Macrophage scavenger receptors take up oxidized low-density lipoprotein to promote cholesterol ester accumulation and foam cell formation, which is another factor contributing to atherosclerotic disease. In animal models, quercetin attenuated atherosclerosis by interfering with foam cell formation and pro-oxidant/proinflammatory response, which were key proatherogenic activities of macrophages [45].

Polyphenols could also protect the cardiovascular system, not only from oxidative stress but other damage because they possess other physiological effects, such as blood pressure reduction and inflammation decreasing action [46]. Dehydroglyasperin C (an antioxidant compound of liquorice) attenuated proliferation and migration of human arterial smooth muscle cells, which was induced by platelet-derived growth factor [47]. Since accumulation of vascular smooth muscle cells plays a key role in the formation and development of lesions in atherosclerosis, dehydroglyasperin C may be beneficial to CVD. In addition, a study showed that seven phenolic acids from blueberries exerted potential atheroprotective effects [48]. Platelet aggregation and adhesion under pathophysiological conditions can cause thrombosis and blockage of coronary arteries, which is associated with the pathogenesis and severity of CVD. Antioxidant polyphenols could modify molecular events towards inhibition of platelet aggregation [21]. Stilbenoids (isolated from *Gnetum macrostachyum*), which possessed antioxidant and anti-inflammation activities, showed inhibitory effects on human platelet aggregation and adhesion [49]. In addition, phlorizin (a polyphenol found in apple) possessed strong antioxidant activity, and prevented diabetic macrovascular complications in diabetic mice [50]. Since diabetic macrovascular complications are important causes of CVD, phlorizin may be beneficial. Furthermore, anthocyanins were also demonstrated to have protective action against several cardiovascular risk factors [51].

Except for polyphenols, other antioxidant phytochemicals such as crocin, lycopene and allicin also show protective activities for CVD. Crocin, a natural antioxidant carotenoid, inhibited platelet aggregation and protected oxidative stress-induced apoptosis of platelets [52]. In addition, several studies indicated that people consuming a Mediterranean diet with high amounts of tomato products had a lower cardiovascular disease risk because lycopene could improve endothelial function of CVD patients [53,54]. Furthermore, allicin, an antioxidant organosulfur compound from garlic, was found to protect the cardiovascular system by inducing vasorelaxation and alleviating cardiac hypertrophy, angiogenesis, platelet aggregation, hyperlipidemia and hyperglycemia [55]. Therefore, antioxidant phytochemicals could be good candidates for preventing and treating CVD through direct antioxidant activity as well as their other bioactivities (such as anti-inflammation and preventing platelet aggregation and adhesion).

### 3.2. Anti-Obesity Activity

The incidence of obesity is rising all around the world and becoming a major public health burden with enormous social and economic costs. Obesity is often accompanied by an increased risk of mortality, and the quality of life is impaired owing to sleep apnea, respiratory problems, osteoarthritis, and infertility [56]. In addition, antioxidant defenses (endogenous antioxidant compounds and antioxidant enzymes) of the obese are lower compared with their normal weight counterparts, which is inversely correlated to central adiposity [57]. Low-grade chronic inflammation induced by inflammatory factors, such as tumor necrosis factor-α, interleukin-6 and monocyte chemotactic protein-1, is another key factor in the pathogenesis of obesity, which may act synergistically with oxidative stress to induce obesity. Chronic inflammation begins in white adipose tissue and eventually becomes systemic in obesity and T2D.

Fruits and plant extracts with high contents of antioxidant phytochemicals show anti-obesity activities both *in vitro* and *in vivo*. For example, citrus fruits showed potential inhibitory activity on α-glucosidase and pancreatic lipase *in vitro*, due to their high contents of antioxidant phytochemicals such as flavanones [58]. In addition, phytochemicals of *Vaccinium floribundum* and *Aristotelia chilensis* including anthocyanins and proanthocyanidins limited adipogenesis and inflammatory pathways *in vitro* [59]. Furthermore, a diet-induced obesity model was reversed by treatment with *Plantago maxima* extract, which contained a high amount of antioxidant compounds including flavonoids, iridoids, phenol carboxylic acids, tannins and ascorbic acid [60]. Obesity-mediated chronic inflammation was reduced by polyphenol-rich grape products through multiple mechanisms, such as antioxidant action, attenuating endoplasmic reticulum stress signaling, blocking proinflammatory cytokines, suppressing inflammatory- or inducing metabolic-gene expression via increasing histone deacetylase activity, and activating transcription factors that antagonize chronic inflammation [61]. Therefore antioxidant phytochemicals are of certain importance for obesity, especially those with anti-inflammatory activity.

Several flavonoids isolated from fruits and plant extracts showed potent anti-obesity activity *in vitro* and *in vivo*. For example, genistein was reported to regulate adipocyte life-cycle and lower obesity-related low-grade inflammation and oxidative stress [62]. In addition, quercetin reduced high fat dietary-induced body weight gain in mice, and also improved insulin sensitivity and glucose intolerance [63]. The anti-adipogenesis activity of quercetin may be mediated by the adenosine monophosphate-activated protein kinase (AMPK) and mitogen-activated protein kinases signaling pathways (MAPK), respectively in preadipocytes and mature adipocytes [64]. Furthermore, naringenin, luteolin and kaempferol also showed anti-adipogenesis activities [65].

Several other phytochemicals also possess anti-obesity activity. For example, resveratrol inhibited adipogenesis to exert its anti-obesity action *in vitro* [66]. In addition, caffeic acid and chlorogenic acid significantly lowered body weight, visceral fat mass and plasma leptin as well as insulin levels in high fat induced-obese mice compared to the control group, while chlorogenic acid had more potent than caffeic acid [67]. Furthermore, curcumin reduced obesity and curtailed the adverse health effects of obesity [68], and allicin also exerted anti-adipogenesis activities [65]. Therefore, antioxidant phytochemicals can be good candidates for the prevention and treatment of obesity through direct inhibition of adipogenesis as well as anti-inflammation and antioxidative action.

### 3.3. Anti-Diabetes Activity

Diabetes is a major worldwide health problem and characterized by chronic hyperglycaemia which leads to a number of microvascular and macrovascular complications (e.g., endothelial dysfunction and atherosclerosis). There are two types of diabetes, type-1 diabetes (T1D) and T2D. Diabetes is usually accompanied by increased production of free radicals or oxidative stress due to hyperglycemia and hyperlipidemia [6]. It was also demonstrated that, in the course of diabetes and its complications, plasma antioxidants including α- and γ-tocopherol, α- and β-carotene, lycopene, β-cryptoxanthin, lutein, zeaxanthin, retinol and ascorbic acid showed a significant decrease [69]. Cohort studies showed that metabolic homeostasis was improved, and the development of T2D and its complications was delayed or prevented by frequent consumption of wholegrain foods [70]. A study showed that antioxidant activities of *Ascophyllum nodosum* (a brown seaweed) were correlated with the phenolic contents, while the α-glucosidase inhibitory activity exhibited a pattern similar to the phenolic contents observed *in vitro* [71]. Thus, the anti-diabetic action might be due to the antioxidantive effect. Another study showed similar results [72]. Lime is a candidate for antidiabetic and antilipolytic purposes (e.g., α-glucosidase and lipase inhibition), which were correlated with flavone contents. In addition, berry polyphenols from maqui and blackthorn may offer dietary and convenient alternatives to control hyperglycaemia in diabetes [72].

Major polyphenolic constituents of *Lactuca sativa* were associated with antidiabetic activity in mice [73]. Another study indicated that the aqueous extract of *Chrysobalanus icaco* showed strong antioxidant action and reduction of glycemia in rats [6]. Additionally, the juices from raphanus, ajwain, sowa and amaranthus leaves were potent source of biological antioxidants, and possessed capacity to mitigate starch induced postprandial glycemic burden and reduced glucose induced postprandial glycemic excursion in rats [74]. Besides, *Aloe greatheadii* contained a variety of antioxidant phytochemicals, and was demonstrated to have therapeutic actions for diabetes by lowering blood glucose [75].

Sub-clinical grade inflammation also plays a significant role in the development of obesity-related insulin resistance. Polyphenols found in grape products reduced obesity-mediated chronic inflammation to prevent metabolic diseases. They act as an antioxidant, blocking proinflammatory cytokines or endotoxin-mediated kinases and transcription factors to exert its antidiabetic activity [61]. In addition, curcumin was considered suitable for the prevention and amelioration of diabetes due to its anti-inflammatory and anti-oxidant activities [76]. Furthermore, butein, an antioxidant polyphenol, inhibited formation of nitric oxide *in vitro*, thus protecting pancreatic β-cells against cytokine-induced toxicity, and could be used for preventing the progression of T1D [77].

Resveratrol may influence expression of genes relevant to the development of T2D, such as by inducing expression of several β-cell genes and insulin expression in pancreatic α-cells [78]. Another study showed that ferulic acid had hypoglycemic activity in streptozotocin induced diabetic rats, and also had a synergistic action with hypoglycemic drugs [79]. In addition, kaempferol was found to promote β-cell survival, improve insulin secretory function and ameliorate hyperglycemia [80], and daidzein could promote glucose uptake and improves glucose homeostasis in the model mice of T2D [81]. Furthermore, naringenin was found to act as an antioxidant and improve diabetes-induced memory dysfunction by inhibition of elevated cholinesterase activity in T2D rats [82], and genistein could prevent diabetes [83]. Especially, supplementation with pycnogenol (PYC), which is a blend of procyanidins comprising of catechin and epicatechin subunits with varying chain lengths, has been shown to lower glucose levels, suggesting anti-diabetes activity in T2D patients [84]. In conclusion, phytochemicals could prevent diabetes through regulating α-glucosidase and lipase activities, reducing postprandial glycemic level, anti-inflammatory activity, improving pancreatic function and synergistic action with hypoglycemic drugs.

### 3.4. Anti-Cancer Activity

Free radicals are considered to be involved in the multistage carcinogenic process. Peroxyl radicals and lipid peroxidation can independently cause mutations on DNA, which are crucial for the initiation of the carcinogenic process. Antioxidant phytochemicals may modulate the initiation of carcinogenesis process by protecting against DNA damage. Recent studies have suggested that the appropriate lifestyle modifications could prevent more than two-thirds of human cancers and the diet contributes to about 35% of human cancer mortality [85]. Consumption of fruits and vegetables was shown to be inversely related to a variety of cancers. An animal study showed the antioxidant and free radical quenching property of the phytochemicals in ethanol extract of *Amaranthus paniculatus* could contribute to its antitumor effect [86]. Polyphenols play an important role in anti-cancer activity of phytochemicals. For instance, polyphenols ellagitannins and epicatechin gallate showed anticarcinogenic properties [87,88]. It has been suggested that biophenols showed toxicity to rapidly proliferating cancer cells but were not toxic to normal cells [89]. Furthermore, green tea polyphenols, silymarin from milk thistle, and proanthocyanidins from grape seeds, had the ability to protect the skin from the adverse effects of UV radiation, such as the risk of skin cancers [90]. The protective property was mainly through four mechanisms, against UV radiation-induced inflammation, oxidative stress, DNA damage, and suppression of immune responses.

Antioxidant phytochemicals could inhibit cell proliferation and induce cancer cell death. Curcumin targeted cancer stem cells to express its anticancer ability [91], and it could exert synergistic anticancer activity with catechin against human colon adenocarcinoma HCT 15, HCT 116, and human larynx carcinoma cell lines [92]. Yang and Liu [93] found that quercetin, genistein, and resveratrol exhibited higher induction of quinone reductase than other compounds among the 18 phytochemicals tested. Upregulation of quinone reductase is thought to be a useful biomarker for anticarcinogenesis. The anticancer effects of quercetin in a variety of cancers, such as prostate, breast, colon, and lung cancers were demonstrated *in vitro* and *in vivo* assays [85], and resveratrol exerted anticancer action by inhibiting tumor initiation, promotion, and progression [94,95]. Additionally, butein showed anti-cancer activity by inducing cell-cycle arrest and apoptosis in human lung cancer cells [96]. Furthermore, lycopene intake and serum lycopene levels were inversely related to certain types of cancers including breast, colon, tongue, gastric and other organ sites [97,98,99,100], and lycopene and β-carotene could inhibit cell proliferation, arrest cell cycle, increase apoptosis of human breast cancer cells [101]. Besides, linalool also showed anticancer action [102].

Especially, antioxidant phytochemicals may downregulate the expression of cancer-related genes to exert anticancer activity, such as cyanidin, keampferol and genistein [103,104]. Cyanidin and keampferol possessed potential ability to downregulate cyclooxygenase 2 (COX-2) gene expression and subsequent gene products related with cancer. Increased activity of COX-2 is often observed in a variety of cancers including breast, prostate, and lung cancers [103]. Genistein showed anticancer effect on breast cancer by demethylating and reactivating methylation-silenced tumor suppressor gene, which were through directly interacting with the catalytic domain of DNA methyltransferase-1 and inhibiting the expression of DNA methyltransferase-1 [104].

### 3.5. Anti-Aging Activity

Aging is commonly related to functional decline of the organism and progressive deleterious alterations leading to increased risk of disease and death with advancing age [105]. Aging also presents motor and cognitive deficits. Free radicals and oxidative stress have been considered as important factors in the biology of aging and in many age-associated degenerative diseases, since the antioxidant systems are under deterioration during aging [106]. Antioxidant phytochemicals have the potential to fight against aging and its related disorders. For example, coffee, which contains high levels of antioxidant phytochemicals, reduced both motor and cognitive deficits in aged rats [107], and methanol extract of *Elaeis guineensis* leaves with high antioxidant activities also showed potential ability as an anti-aging agent [108]. In addition, antioxidant phytochemicals showed anti-ageing activities by different mechanisms. For example, epigallocatechin gallate (EGCG) extended lifespan of healthy rats by reducing the damage of liver and kidney and improving age-associated inflammation and oxidative stress through inhibiting NF-κB signaling [109], and tetrahydroxystilbene glucoside showed protective effect against the d-galactose-induced aging process by regulating Klotho gene in mice [110]. Another study found that allicin could significantly ameliorate cognitive dysfunction in aged mice through enhancing of nuclear factor-like 2 antioxidant signaling pathways [111]. Furthermore, curcumin, resveratrol, and proanthocyanidins protected age-related cognitive decline and depression by modulating hypothalamic-pituitary-adrenal axis activity, serotonergic transmission and hippocampal neurogenesis [112]. In a word, phytochemicals could extend lifespan, ameliorate cognitive dysfunction and improve age-associated inflammation and oxidative stress.

### 3.6. Protective Action on Alzheimer’s Disease

Alzheimer’s disease (AD) is a degenerative neurological disorder characterized by cognitive decline and memory loss [113]. The brain is believed to be particularly vulnerable to oxidative stress due to a relatively high concentration of oxygen free radicals without commensurate levels of antioxidative defenses. Oxidative stress may be involved in pathogenesis of dementia or AD in elderly [114]. AD patients show a remarkable reduction of acetylcholine (ACh) levels in the hippocampus and cortex of the brain, which can cause memory deficits. Acetylcholinestrase inhibition is linked to amelioration of Alzheimer’s symptoms.

A population-based study showed that dietary flavonoids were associated with lower population rates of dementia in European countries, New Zealand, Australia, USA and Canada [115]. This effect was mainly dependent on antioxidant phytochemical-mediated reduction of oxidant stress and acetylcholinestrase in the brain. Another study indicated that quercetin possessed protective effects against neurotoxicity of amyloid β-peptide, which was elevated in AD brain, by modulating oxidative stress at low dose *in vitro* [116]. In addition, the extracts of *Crataegus pinnatifida* fruit showed potential neuroprotective activity for preventing oxidative-related disorders *in vitro* [117], and the ethyl acetate extract of *Aegle marmelos* showed antioxidant activity as well as potential acetylcholinestrase inhibitory property [118]. Furthermore, the chokeberry concentrate and lemon juice showed high antioxidant effect and inhibition of cholinesterase [119].

The flavonoids (including naringenin, hesperetin, eriodictyol and their derivatives) from *Paulownia tomentosa* fruit exhibited a significant inhibition of both acetylcholinestrase and butyrylcholinestrase [120]. In addition, curcumin, catechins, and resveratrol showed neuroprotective ability in AD [121], and curcumin (200 and 400 mg/kg) treatment reduced levels of oxidative stress in a dose dependent manner as well as attenuated increased acetylcholinesterase in mice [122]. Furthermore, polyphenolic compounds of walnuts reduced the oxidant and inflammatory load on brain cells, improved interneuronal signaling, increased neurogenesis, as well as enhanced sequestration of insoluble toxic protein aggregates [27], so walnuts could play a role in preventing AD. Therefore, phytochemicals could protect against AD by reduction of oxidant stress and acetylcholinestrase.

### 3.7. Protective Action on Inflammatory Bowel Disease

Inflammatory bowel disease (IBD), which includes two complex diseases, ulcerative colitis (UC) and Crohn’s disease, is a chronic inflammatory disorder caused by deregulated immune responses in a genetically predisposed individual [123]. Antioxidant phytochemicals were highly associated with IBD, because quite a lot of them were found to have anti-inflammatory properties. In addition, it was shown that patients with UC often had antioxidant nutrient deficiencies at the time of diagnosis [124]. Polyphenol-enriched cocoa extract (PCE) with epicatechin, procyanidin B2, catechin, and procyanidin B1 as the major phenolics showed anti-inflammatory properties against dextran sulfate sodium (DSS)-induced UC in mice. PCE significantly reduced both the extent and the severity of the inflammation as well as the crypt damage and leukocyte infiltration in the mucosa, which resulted in reducing colon damage [125]. Another study showed that dietary supplementation with 10% cattail rhizome flour and its combination with prednisolone prevented the trinitrobenzenesulphonic acid (TNBS)-induced colitis in rats, but no synergistic effects were observed. In addition, the antioxidant properties of the active compounds in the cattail rhizome resulted in an improvement in intestinal oxidative stress, which was associated with the prevention of TNBS-induced colon damage [126]. Furthermore, the antidiarrheal, gastroprotective and cicatrising effects of *Hymenaea stigonocarpa* could be associated with the antioxidant effect [127,128]. Besides, curcumin has shown potential efficacy in ulcerative colitis patients, and antioxidant phytochemicals could directly suppress inflammatory responses in the course of UC development [124]. These studies showed antioxidant phytochemicals could possess protective actions for IBD through anti-inflammation.

### 3.8. Other Biological Activities

Antioxidant phytochemicals could possess protective actions against many other chronic diseases besides the diseases mentioned above. Chinese medicinal plants showed strong antioxidant activities in the treatment of rheumatic diseases, and the antioxidant phytochemicals in them could be effective components [129]. Another study showed that lutein and zeaxanthin may be beneficial for cataract and macular degeneration (two common eye diseases of aging) [130]. In addition, some antioxidant flavonoids have antihypertensive effects, such as quercetin and morin, in animal and human studies [46,131,132]. Furthermore, some antioxidant phytochemicals and their target chronic diseases are summarized in Table 1, while some antioxidant phytochemicals which were proved to have preventive and/or therapeutic effect by human studies are summarized in Table 2.

**Table 1:** Antioxidant phytochemicals and their target chronic diseases.

Classification	Antioxidants	Chronic Disease	Reference
carotenoids	crocin	CVD	[52]
	lutein	diabetes;	[69]
		cataract; macular degeneration	[130]
	lycopene	CVD;	[53]
		diabetes;	[69]
		cancer	[97,98,99,100,101]
	zeaxanthin	cataract; macular degeneration	[130]
flavonoids	anthocyanins	CVD	[51]
	dehydroglyasperin C	CVD	[47]
	phloretin	diabetes; CVD	[50]
flavonols	keampferol	obesity;	[65]
		diabetes;	[80]
		cancer;	[103]
	morin	hypertension	[46]
	quercetin	CVD;	[45]
		obesity;	[63]
		cancer;	[85]
		AD;	[116]
		hypertension	[132]
flavanols	catechin	diabetes;	[84]
		cancer;	[92]
		AD	[121]
	epicatechin	diabetes	[84]
	epicatechin gallate	cancer	[88]
	epigallocatechin gallate	aging	[109]
flavanones	eriodictyol	AD	[120]
	hesperetin	AD	[120]
	naringenin	obesity;	[65]
		diabetes;	[82]
		AD	[120]
flavones	luteolin	obesity	[65]
isoflavonoids	daidzein	diabetes	[81]
	genistein	obesity;	[62]
		diabetes;	[83]
		cancer	[104]
organosulfur compounds	allicin	CVD;	[55]
		obesity;	[65]
		aging	[111]
phenolic acids	caffeic acid	obesity	[67]
	chlorogenic acid	obesity	[67]
	ferulic acid	diabetes	[79]
polyphenols	butein	diabetes;	[77]
		cancer	[96]
	curcumin	ulcerative colitis;	[124]
		obesity;	[68]
		diabetes;	[76]
		cancer;	[92]
		aging;	[112]
		AD	[122]
	resveratrol	CVD;	[40]
		obesity;	[66]
		diabetes;	[78]
		cancer;	[94,95]
		aging;	[112]
		AD	[121]
stilbenes	tetrahydroxystilbene glucoside	aging	[110]
tannins	ellagitannins	cancer	[87]

**Table 2:** Antioxidant phytochemicals showing preventive and/or therapeutic effect by human studies.

Phytochemicals	Disease	Reference
lycopene	improved endothelial function in CVD patients on optimal secondary prevention	[53,54]
allicin	reduced total blood cholesterol, LDL and raised HDL	[55]
pycnogenol	resulted in improved diabetes control, lowered CVD risk factors, and reduced antihypertensive medicine use	[84]
flavonols	associated with lower population rates of dementia in European countries, New Zealand, Australia, USA and Canada at a large-scale population level	[115]
curcumin	potential therapeutic capability in UC patients	[124]
flavonoids	contributed to the prevention of hypertension in a prospective study in men and women	[131]
quercetin	reduced blood pressure in stage I hypertensive patients	[132]

## 4. Bioavailability

The concept of bioavailability was introduced to quantify the amount of micronutrients and phytochemicals that is actually absorbed, distributed to the tissue, metabolized and eventually excreted. Bioavailability is defined as “the rate and extent to which the therapeutic moiety is absorbed and becomes available to the site of drug action” [133]. Blood of fifteen healthy volunteers was collected at time 0 and 1, 2, 3 and 4 h after consumption of 144 g of raisins. Seventeen phytochemicals including 16 phenolics and oleanolic acid were identified and quantified in volunteers’ plasma. The results indicated that phytochemicals in raisins were bioavailable [134]. In addition, the lycopene concentration of plasma changed from 0.60 ± 0.22 to 1.24 ± 0.30 μmol/L during eight weeks of daily consumption of a soy germ-fortified tomato juice (300 mL supplying 66 mg isoflavones and 22 mg lycopene) in eighteen healthy men and women. Juice consumption also significantly improved resistance of LDL + VLDL-C to Cu^2+^-mediated oxidation, HDL-C, and the ratio of total-C/HDL-C at week 8 [135]. Similar results were also found in old adults [136].

However, some phytochemicals show low levels of solubility, stability, bioavailability, and target specificity in the body, which makes these compounds unrealistic to be present at their effective levels in the target tissues. This is particularly true for EGCG, resveratrol, curcumin and quercetin [137]. For example, the areas under the concentration-time curve (AUC) for EGCG was 39.6 ± 14.2 μg·h/L via oral administration, and was 2772 ± 480 μg·h/L after intravenous administration (10 mg/kg), showing very low bioavailability, as a result of EGCG’s rapid degradation in body fluids [138]. Quercetin also has low aqueous solubility and bioavailability and is quickly metabolized in the body, which may reduce its effect on preventing and treating diseases [139]. In healthy participants, the maximal plasma concentrations (*C*_max_) of quercetin was 0.16 μM after ingesting grape juice containing 10 mg quercetin aglycone, which represents only about 1.4% of the ingested dose [140]. Around 6% of the ingested dose was recovered in plasma and tissues of rats following ingestion of a meal containing radiolabeled quercetin glucoside [141]. Another study showed that the maximal plasma concentrations of resveratrol was about 10 ng/mL, 0.5–2 h after the oral dose treatment of resveratrol with 25 mg, meanwhile, the plasmatic concentrations of resveratrol plus total metabolites were around 400–500 ng/mL, indicating a very low oral bioavailability of free resveratrol [140,142,143]. In addition, it was observed the average peak serum concentrations after taking 4 mg, 6 mg and 8 mg/day of curcumin were 0.51 ± 0.11 μM, 0.63 ± 0.06 μM and 1.77 ± 1.87 μM respectively, which showed the extremely low serum levels [144]. Different pharmaceutical strategies are adopted to increase curcumin bioavailability, such as co-administration of the alkaloid piperine and other compounds, phytosome technology and nanoparticles [145]. For example, curcumin bioavailability increased 20 times in human and 1.56 times in rats when 2 g of curcumin was given with 20 mg of piperine [146]. Nanoparticles of other phenolic phytochemicals could enhance their absorption and bioavailability as well [147]. The bioavailability of polyphenolics can also be improved using PHYTOSOME^®^ delivery system [148].

## 5. Adverse Effects of Antioxidant Phytochemicals

Although many studies have found protective roles for antioxidant phytochemicals in chronic diseases, other studies found some discrepancies. For example, fruit juices from red grape, strawberry, cherry or sour cherry showed very strong free radical scavenging activity in the 2,2-diphenyl-1-picrylhydrazyl radical scavenging capacity assay and the β-carotene bleaching assay, but they did not show cytotoxic effects on HT29 cells using the same concentrations. That is, there was no correlation between the antioxidant activity and anti-proliferative effects in HT29 cells of these fruit juices [149]. In another study, effects of several antioxidant phytochemicals on the tumor promoting activity of 3,3′,4,4′-tetrachlorobiphenyl were examined *in vivo*. Coenzyme 10 increased cell proliferation in normal hepatocytes, whereas the other antioxidants (e.g., ellagic acid, β-carotene, curcumin, N-acetyl cysteine, resveratrol, lycopene, and a tea extract) had no effect in either normal or PGST-positive hepatocytes. The results showed that none of the antioxidant phytochemicals produced a clear decrease in the tumor promoting activity of 3,3′,4,4′-tetrachlorobiphenyl in rats [150]. In addition, it was found by a systematic evaluation that the concentrations of vitamin C, vitamin E, α-carotene and β-carotene in dietary were inversely associated with gastric cancer risk, while no such association was observed for blood levels of these antioxidants [151]. Although some epidemiological studies showed a relationship between the low incidence of cancer and the intake of plant-based foods, at present there is no conclusive proof that high antioxidant activity would result in high anticancer activity [152].

Some studies have also indicated that supplemental antioxidants cannot decrease the risks for some diseases and could even play an inverse role. To assess the effect of antioxidant supplements on mortality in randomized primary and secondary prevention trials, the effect of antioxidant supplements on all-cause mortality was analyzed with random-effects meta-analyses and reported as relative risk with 95% confidence intervals. Meta-regression was used to assess the effect of covariates across the trials. The results showed that vitamin C and selenium had no significant effect on mortality; treatment with β-carotene, vitamin A, and vitamin E may increase mortality [153]. In addition, a review paper also pointed out that the majority of the studies did not support a possible role of antioxidant supplementation in reducing the risk of cardiovascular disease [154]. Thus, the application of antioxidant therapy in certain diseases needs long-term investigations on large-scale cohorts [155].

## 6. Conclusions and Prospects

Chronic diseases are the leading causes of death and disability. Reactive oxygen or nitrogen species under certain conditions can cause an imbalance and lead to oxidative damage to large biomolecules such as lipids, DNA and proteins. Overproduction of oxidants and chronic inflammation are responsible for the pathogenesis of many chronic diseases. Thus, antioxidant phytochemicals are among the most potential agents to treat chronic diseases. They possess many biological activities and health benefits, such as antioxidant and free radical scavenging abilities, anti-inflammatory action, anticancer, anti-aging, and protective action for cardiovascular diseases, diabetes mellitus, obesity and neurodegenerative diseases. Especially, many antioxidant phytochemicals are found to have more than one property, for example, resveratrol has a protective role in CVD, cancers, aging, obesity, diabetes and AD. It is recommended to consume fruits, vegetables, and grains as well as some medicinal plants more frequently because they contain many antioxidant phytochemicals. In the future, more antioxidant phytochemicals in foods and medicinal plants should be separated and identified, and their bioactivities and the mechanism of action should be studied further. In addition, attention should be paid to the potential adverse effects of antioxidant phytochemicals for human beings.
